# Transcriptome analysis during axillary bud growth in chrysanthemum (*chrysanthemum*×*morifolium*)

**DOI:** 10.7717/peerj.16436

**Published:** 2023-12-15

**Authors:** Yijun Chen, Qin Ling, Xin Li, Qiqi Ma, ShaoKang Tang, Pan Yuanzhi, Qing-lin Liu, Yin Jia, Xue Yong, Beibei Jiang

**Affiliations:** College of Landscape Architecture, Sichuan Agricultural University, Chendu, Sichuan, China

**Keywords:** *Chrysanthemum morifolium*, Axillary bud, *DgLsL*, Transcriptome, Differentially expressed genes

## Abstract

The chrysanthemum *DgLsL* gene, homologous with tomato *Ls*, is one of the earliest expressed genes controlling axillary meristem initiation. In this study, the wild-type chrysanthemum (CW) and *DgLsL*-overexpressed line 15 (C15) were used to investigate the regulatory mechanism of axillary bud development in chrysanthemum. Transcriptome sequencing was carried out to detect the differentially expressed genes of the axillary buds 0 h, 24 h and 48 h after decapitation. The phenotypic results showed that the number of axillary buds of C15 was significantly higher than CW. A total of 9,224 DEGs were identified in C15-0 *vs.* CW-0, 10,622 DEGs in C15-24 *vs.* CW-24, and 8,929 DEGs in C15-48 *vs.* CW-48.GO and KEGG pathway enrichment analyses showed that the genes of the flavonoid, phenylpropanoids and plant hormone pathways appeared to be differentially expressed, indicating their important roles in axillary bud germination. *DgLsL* reduces GA content in axillary buds by promoting GA2ox expression.These results confirmed previous studies on axillary bud germination and growth, and revealed the important roles of genes involved in plant hormone biosynthesis and signal transduction, aiding in the study of the gene patterns involved in axillary bud germination and growth.

## Introduction

Chrysanthemum (*Chrysanthemum* × *morifolium*) has a high ornamental value and is one of the four main fresh-cut flowers types worldwide. There are only a few chrysanthemum varieties with little to no axillary buds, and single-headed cut chrysanthemums require a lot of labor and material resources to manually remove axillary buds. This study lays a theoretical foundation for the molecular mechanism of lateral branch development in chrysanthemums and strain genetic improvement, and provides an important theoretical basis for the standardized production of chrysanthemum.

The branch development process is closely related to plant stature, yield, and quality (Du et al., 2017; Holalu & Finlayson, 2017; Dierck et al., 2018). This process starts with axillary meristem initiation followed by the development and formation of flowers or lateral branches (Wang, Smith & Li, 2018), which consists of two key stages: axillary meristem formation and axillary bud growth (Holalu & Finlayson, 2017). In plants, axillary buds are dormant due to the apical buds of the stem, indicating ‘apical dominance.’ Axillary buds meristem activity is genetically controlled and regulated by plant hormones. Previous studies revealed, and the current study verified that auxin, cytokinin (CK), gibberellins (GAs) and strigolactone (SL) are the most important hormones regulating lateral branch growth and development (Pellegrineschi et al., 2004; Raatz et al., 2011). Auxin inhibits the germination and growth of axillary buds. Early studies suggested that auxin is produced by apical buds, then transported from the top to the axillary buds *via* polar transport (Thimann, Skoog & William, 1934). However, later research found that auxin does not enter the axillary buds directly, but affects the growth of axillary buds indirectly through other pathways (Prasad et al., 1993; Booker, Chatfield & Leyser, 2003). Low expression levels of genes related to the flavonoid biosynthetic pathway lead to a decrease in growth hormone transporter proteins in shoots and stems, reducing the transport rates of polar growth hormones (Lazar & Goodman, 2006). Shi et al. (2021) found that in the absence of flavonoids, which act as inhibitors of growth hormone transport, there was an increase in growth hormone accumulation, which in turn induced growth hormone export to promote axillary bud growth. CK is related to plant meristem development, and is capable of promoting axillary bud growth (Morris et al., 2005; Müller & Leyser, 2011). GAs also regulate the growth of axillary buds, but have different effects in different species. In peas, rice and barley, GAs inhibit axillary bud development (Scott, Case & Jacobs, 1967; Lo et al., 2008; Jia et al., 2011; Qi et al., 2011; Luisi, Lorenzi & Sorce, 2011), and in citrus, goldenrod, sweet cherry and moonflower, GAs promotes the growth of axillary buds (Elfving, Visser & Henry, 2011; Choubane et al., 2012).

Genetic studies and genomic analyses have begun to reveal the gene regulatory network (GRN) of axillary meristem (AM) initiation. For example, *CUC* regulates *LAS* expression (Hibara et al., 2006; Raman et al., 2008), and CUC proteins bind directly to the *LAS* promoter to activate *LAS* expression (Tian et al., 2014). Cell-specific expression analyses as well as genomic yeast monohybrid experiments have shown that *CUC2* and *LAS* function as two centers of GRN and their promoter regions are linked to many transcription factors (Tian et al., 2014). For example, *RAX1* and *RAX3* directly activate *CUC2*, then *CUC2* directly activates the miRNA gene miR164, which degrades *CUCI* and *CUC2* transcripts, forming a feedback loop (Mallory et al., 2004; Laufs et al., 2004; Larue, Wen & Walker, 2009). *LS* in tomato is a direct homolog of *LAS* in Arabidopsis. In tomato mutant ls plants, although axillary bud formation is normal after flowering, the recessive ls mutation prevents axillary meristem formation during nutritional growth, exhibiting a deficiency of axillary meristem tissue (Groot et al., 1994).Chrysanthemum *DgLsL* is homologous to the *LS* and *LAS* genes and encodes a protein that belongs to the same GRAS protein family (Yang et al., 2005). Huh et al. (2013) suggested that the Ls gene has a conserved role in chrysanthemum axillary meristem initiation and may interact with other temperature-dependent genes. Jiang et al. (2010) transferred the *DgLsL* sense and antisense genes into the chrysanthemum variety ‘Jinghai,’ and showed that the introduction of the sense transformants significantly promoted meristem initiation, and the number of lateral branches was positively correlated with the expression level of the *DgLsL* gene. *LAS* contributes to the specific expression pattern of the GA inactivating enzyme GA2ox4, which forms a low GA cellular ecotone in regions of axillary bud formation. However, increasing GA levels in leaf axils by ectopic expression of the GA biosynthetic enzyme GA20ox2 significantly impairs axillary meristem initiation (Zhang et al., 2020).There are more studies on the effects of transcription factors of the GRAS family such as ls, las and moc1 on lateral branch development, but there are no studies on the upstream and downstream genes of the *DgLSL* gene in chrysanthemum. Identifying genes in the pathway where *DgLsL* is located will help elucidate the mechanism *DgLsL* uses to regulate axillary bud development.

In this study, the wild-type chrysanthemum (CW) and *DgLsL* overexpressed line 15 (C15) were used to investigate the regulatory mechanism of axillary bud development in chrysanthemum. Compared with CW, C15 has more axillary buds, and C15 and CW have the same genetic background and little environmental interference, which makes them ideal for studying the development of the axillary buds of chrysanthemum. Transcriptome sequencing was carried out to detect the differentially expressed genes (DEGs) of the axillary buds at 0 h, 24 h and 48 h after decapitation, in order to study the regulation mode of axillary bud development in the early stage.

## Methods

### Plant materials

Wild-type *Chrysanthemum* × *morifolium* ‘Jinba’ (CW) and *DgLsL* overexpressed line 15 (C15) with height of 8–10 cm were used for this study. The study plants were preserved by the School of Landscape Architecture of Sichuan Agricultural University, and the seedlings were grown in a medium supplemented with 30 g/L sucrose, 7.0 g/L agar and 4.4 g/L MS (Murashige & Skoog, 1962). They were placed in a room with a temperature of 23 °C, a photoperiod of 16 h light/8 h dark, relative humidity of 65% and a light intensity of 100–120 µmol/(m^2^ s). The chrysanthemum was decapitated, and the first axillary bud after decapitation was used as bud 1 and the second axillary bud as bud 2. Bud1 and bud2 (one cm stem segments with buds,) were collected 0 h, 24 h and 48 h after decapitation, and 0 h samples were taken prior to decapitation. The statistical power of this experimental design, calculated in RNASeqPower (v1.38.0) was 0.83. All samples were collected and stored at − 80 °C for RNA-Seq and qRT-PCR sequencing. Resampling to measure the length of lateral bud1 and bud2 0 h, 24 h and 48 h after decapitation for phenotypic recording. Two technical and three biological replicates were performed.

### RNA extraction and sequencing

Total RNA were extracted following the manufacturer of the RNA Prep Pure Plant Kit (Tiangen, Beijing, China). Total amounts and integrity of RNA were assessed using the RNA Nano 6000 Assay Kit of the Bioanalyzer 2100 system (Agilent Technologies, Santa Clara, CA, USA). The test method is the same as previously described in Li et al. (2021). mRNA was purified using poly-T and oligo-attached magnetic beads. First strand and second strand cDNA was synthesized using random hexamer primer and M-MuLV Reverse Transcriptase (RNase H^−^), and DNA Polymerase I and dNTP, respectively. The cDNA fragments of 370∼420 bp in length were selected and purified with AMPure XP system (Beckman Coulter, Brea, CA, USA). PCR amplification was then performed; the PCR product was purified by AMPure XP beads, and the library was finally obtained. The insert size of the library was detected using the Agilent 2100 bioanalyzer. The library preparations were sequenced on an Illumina NovaSeq 6000 platformand and 150 bp pairing is generated.

### Analysis of data

Clean data (clean reads) were obtained by removing reads containing adapters or N base and low-quality reads from raw data. Q20, Q30 and GC content were calculated from the clean date. The Trinity software (v2.6.6) with min_kmer_cov:3 and other  parameters being default accordingly was used to assemble the clean reads for the reference sequence (Grabherr et al., 2011). Hierarchical clustering used Corset (version 4.6) with default parameters, hierarchical clustering of transcripts based on their reads and expression patterns. BUSCO (based on eudicots_odb10.2020-09-10.tar.gz database) software was used to evaluate the splicing quality of Trinity, unigene and cluster, and the accuracy and completeness of the splicing results were evaluated according to the proportion and completeness of the comparison.

Gene function was annotated based on Nr (NCBI non-redundant), Nt (NCBI non-redundant nucleotide sequences), Pfam (Protein family), COG (Cluster of orthologous groups), KOG (euKaryotic Orthologous Groups), Swiss-prot (a manually annotated and reviewed protein sequence database), KEGG (Kyoto Encyclopedia of Genes and Genomes) and GO(Gene Ontology) databases. Differential expression analyses of two conditions/groups (two biological replicates per condition) were performed using the DESeq2 R package (1.20.0); padj < 0.05 and —log2(foldchange)— > 1 were set as the significance threshold for differential expression. GOseq (1.10.0) and KOBAS (v2.0.12) software were used for GO function enrichment analyses and KEGG pathway enrichment analyses of differential gene sets.

### Validation of RNA-Seq by qRT-PCR

To verify the validity of the transcriptome data, nine genes were chosen for qRT-PCR. The extraction and quality detection of total RNA were the same as RNA-seq. Total RNA was converted into cDNA using the TransScript^®^ II All-in-One First-Strand cDNA Synthesis SuperMix for qPCR (One-Step gDNA Removal; TransGen Biotech, Beijing, China), according to the manufacturer’s protocol. Relative expression levels were calculated by the 2^−ΔΔCt^ method, and the *Actin* gene (Forward primer: ACAACTGCTGAACGGGAAAT, Reverse primer: AATCATAGACGGCTGGAAAAG) was used as a reference for quantitative expression analysis. The primers used in qRT-PCR are listed in Table S1. The the reagents and reaction conditions of the reverse transcription PCR (RT-PCR) and qRT-PCR can be found in the supplementary tables (Tables S2–S4).

## Results

### The phenotype of CW and C15

**Figure 1:**
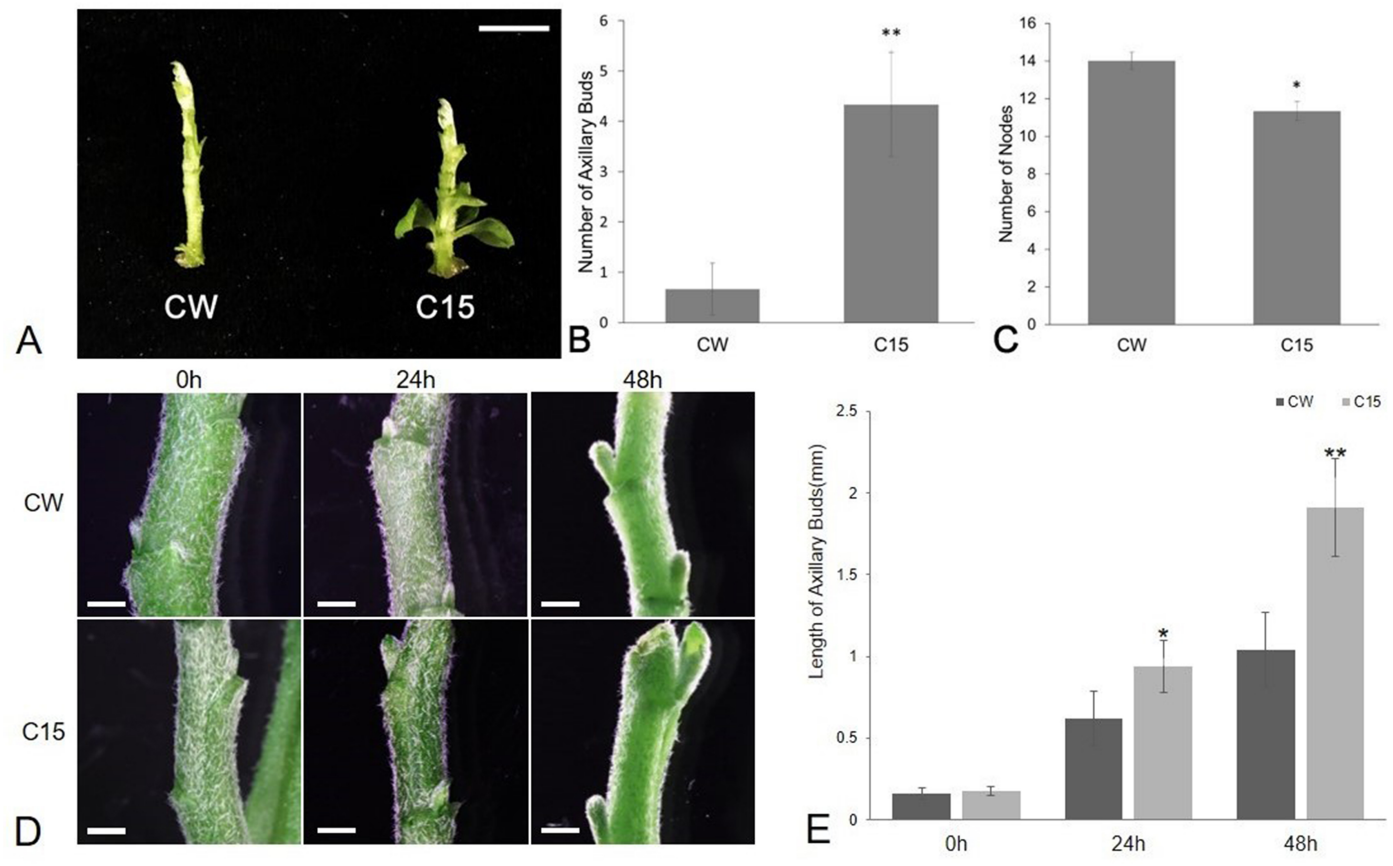
The phenotype analysis of CW and C15. (A) The phenotypes at the same stage. Bar = 1 cm. (B) The number of axillary buds. (C) The number of nodes.

The number of axillary buds and plant nodes in chrysanthemums were counted as shown in Fig. 1. The average number of axillary buds was 0.67 for CW and 4.33 for C15. The number of axillary buds was significantly higher for C15 compared to than CW, and the axillary buds were mostly concentrated in the lower part of the plant (Figs. 1A–1B). C15 had a significant lower average number of plant nodes than CW (11.33 *vs* 14; Fig. 1C). The lengths of bud1 and bud2 were measured at 0, 24 and 48 h after decapitation and then the lengths were averaged. C15 axillary buds were longer than CW’s after decapitation (Fig. 1D). CW and C15 lengths were not significantly different at 0 h; C15 axillary bud lengths were significantly different from CW at 24 h and reached a highly significant difference at 48 h (Fig. 1E). The results were described using standard errors. Statistics and analyses of data were performed using SPSS software (v25.0; SPSS Inc., Chicago, IL, USA).

### RNA sequencing and transcriptome analysis

In total, 413,884,995 raw reads (three replicates per sample) were generated from the CW-0, CW-24, CW-48, C15-0, C15-24 and C15-48 libraries. The percentage of nucleotides with quality scores higher than 20 (Q20) were all above 97%, and the Q30 content ranged from 92% to 94% (Table S5). After filtering the reads, removing quality scores lower than Q20, more than 97.60% of the reads were identified as clean reads. Clean reads were assembled using Trinity (Grabherr et al., 2011), and a total of 149,496 unigenes were obtained. Most of the unigenes were 300–500 bp in length, and the smallest number of unigenes were longer than 2,000 bp (Table S6). The functional annotation of unigenes was performed using the Nr, Nt, Pfam, KOG/COG, Swiss-prot, KEGG, and GO databases (Table S7).

### Identification and analysis of DEGs

**Figure 2:**
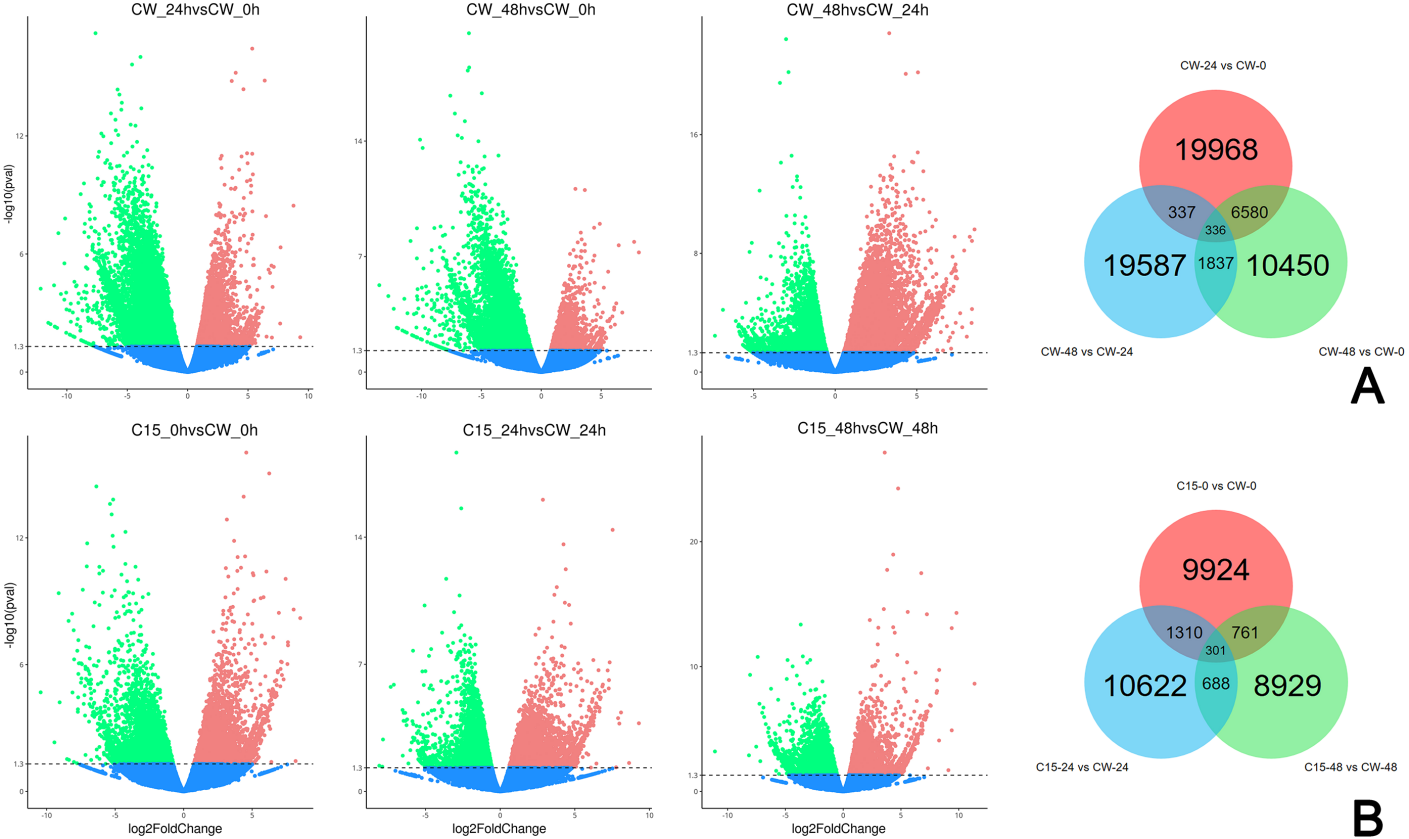
Volcano map and Venn diagrams of DEGs of different stages and strains. (A) DEGs of CW 0 h, 24 h, and 48 h after decapitation. (B) DEGs of C15 *vs.* CW 0 h, 24 h, and 48 h after decapitation. (Red dots indicate up-regulated DEGs, green dots indicate down-regulated DEGs, and blue dots indicate genes that were not differential expressed.).

FPKM values were used to indicate the abundance of each gene. DEGs were defined as having —log2(FoldChange)— > 1 and padj < 0.05. The results showed that many genes were differentially expressed after decapitation. A total of 19,968 DEGs were identified in CW-24 *vs.* CW-0, 10,450 DEGs were identified in CW-48 *vs.* CW-0, and there were 19,587 DEGs in CW-48 *vs.* CW-24. There were 336 DEGs identified between all three comparison groups (Fig. 2A). Meanwhile, there was a total of 9,224 DEGs identified in C15-0 *vs.* CW-0, 10,622 DEGs in C15-24 *vs.* CW-24, and 8,929 DEGs in C15-48 *vs.* CW-48, and these groups had 301 shared DEGs (Fig. 2B).

### Functional classification and pathway analysis of DEGs

To further analyze the differential genes, GO functional classification and an enrichment analysis were performed (Fig. S1). The results showed that in the molecular MF) category, the DEGs were mainly involved in ion binding, ATPase activity and kinase activity. The DEGs annotated in the cellular component (CC) category were mainly related to chromosomes, the cell wall and external encapsulating structure. In the biological processes (BP) category, the DEGs were mainly involved in the cellular protein modification process, ribosome biogenesis, and the cellular protein modification process.

A total of 149,496 unigenes were assigned to 295 pathways based on KEGG annotation. Among these unigenes, 3,152 unigenes were involved in signal transduction, 2,574 genes were involved in translation, and 2,137 unigenes were related to carbohydrate metabolism (Fig. S2). The most significantly enriched pathways in the DEGs of the different comparison groups (padj < 0.05) are shown in Table 1, including phenylpropanoid biosynthesis, flavonoid biosynthesis, photosynthesis and other pathways involved in plant growth and development and plant axillary bud growth.

### qRT-PCR validation

**Figure 3:**
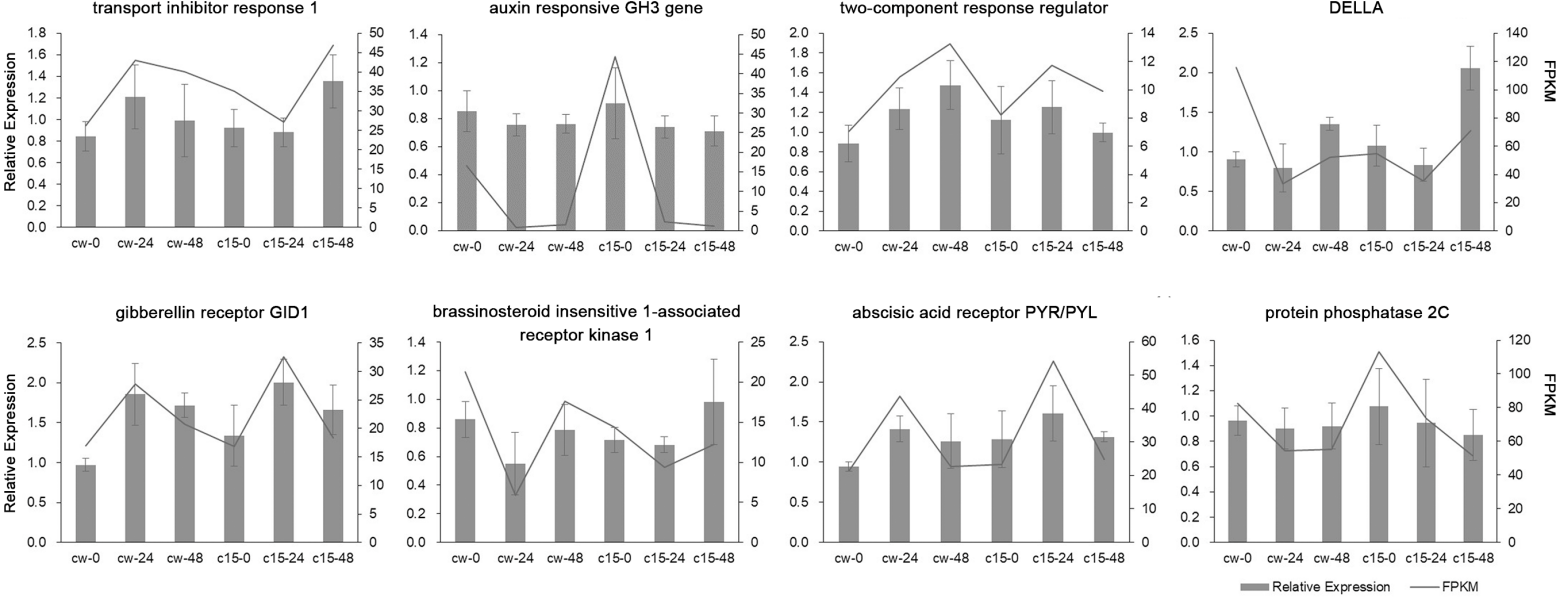
Validation of RNA-Seq data by qRT-PCR analysis.

Eight DEGs belonging to the plant hormone signal transduction pathway were randomly selected for qRT-PCR validation (Fig. 3). The qRT-PCR results of these DEGs were mostly in agreement with the RNA-seq results, which indicated that the RNA-seq results were reliable.

**Table 1:** Pathways of DEGs annotated in KEGG.

NO.	Pathway annotated in KEGG	Count	Up	Down	Pathway ID
CW-24 *vs.* CW-0					
1	Ribosome	282	250	32	ko03010
2	Photosynthesis	33	32	1	ko00195
3	Flavonoid biosynthesis	32	12	20	ko00941
4	Photosynthesis - antenna proteins	25	25	0	ko00196
5	Stilbenoid, diarylheptanoid and gingerol biosynthesis	23	4	19	ko00945
CW-48 *vs.* CW-0					
1	Plant hormone signal transduction	99	19	80	ko04075
2	Phenylpropanoid biosynthesis	90	12	78	ko00940
3	Phenylalanine metabolism	40	4	36	ko00360
4	Flavonoid biosynthesis	28	3	25	ko00941
5	Stilbenoid, diarylheptanoid and gingerol biosynthesis	25	2	23	ko00945
CW-48 *vs.* CW-24					
1	Spliceosome	181	87	94	ko03040
2	Cell cycle	102	76	26	ko04110
3	RNA degradation	95	65	30	ko03018
4	Ribosome biogenesis in eukaryotes	67	49	18	ko03008
5	Photosynthesis - antenna proteins	30	5	25	ko00196
C15-0 *vs.* CW-0					
1	Phenylpropanoid biosynthesis	86	15	71	ko00940
2	Drug metabolism - cytochrome P450	32	4	28	ko00982
3	Phenylalanine metabolism	32	5	27	ko00360
4	Metabolism of xenobiotics by cytochrome P450	30	2	28	ko00980
5	Flavonoid biosynthesis	26	3	23	ko00941
C15-24 *vs.* CW-24					
1	Proteasome	63	5	58	ko03050
2	Terpenoid backbone biosynthesis	53	4	49	Ko00900
3	Photosynthesis - antenna proteins	39	0	39	ko00196
4	Photosynthesis	36	1	35	ko00195
5	Flavonoid biosynthesis	29	11	18	ko00941
C15-48 *vs.* CW-48					
1	Photosynthesis	47	46	1	ko00195
2	Photosynthesis - antenna proteins	46	46	0	ko00196
3	Glyoxylate and dicarboxylate metabolism	41	37	4	ko00630
4	Porphyrin and chlorophyll metabolism	29	25	4	ko00860
5	Flavonoid biosynthesis	17	10	7	ko00941

### Analysis of DEGs in the flavonoid biosynthesis and phenylpropanoid biosynthesis pathways

**Figure 4:**
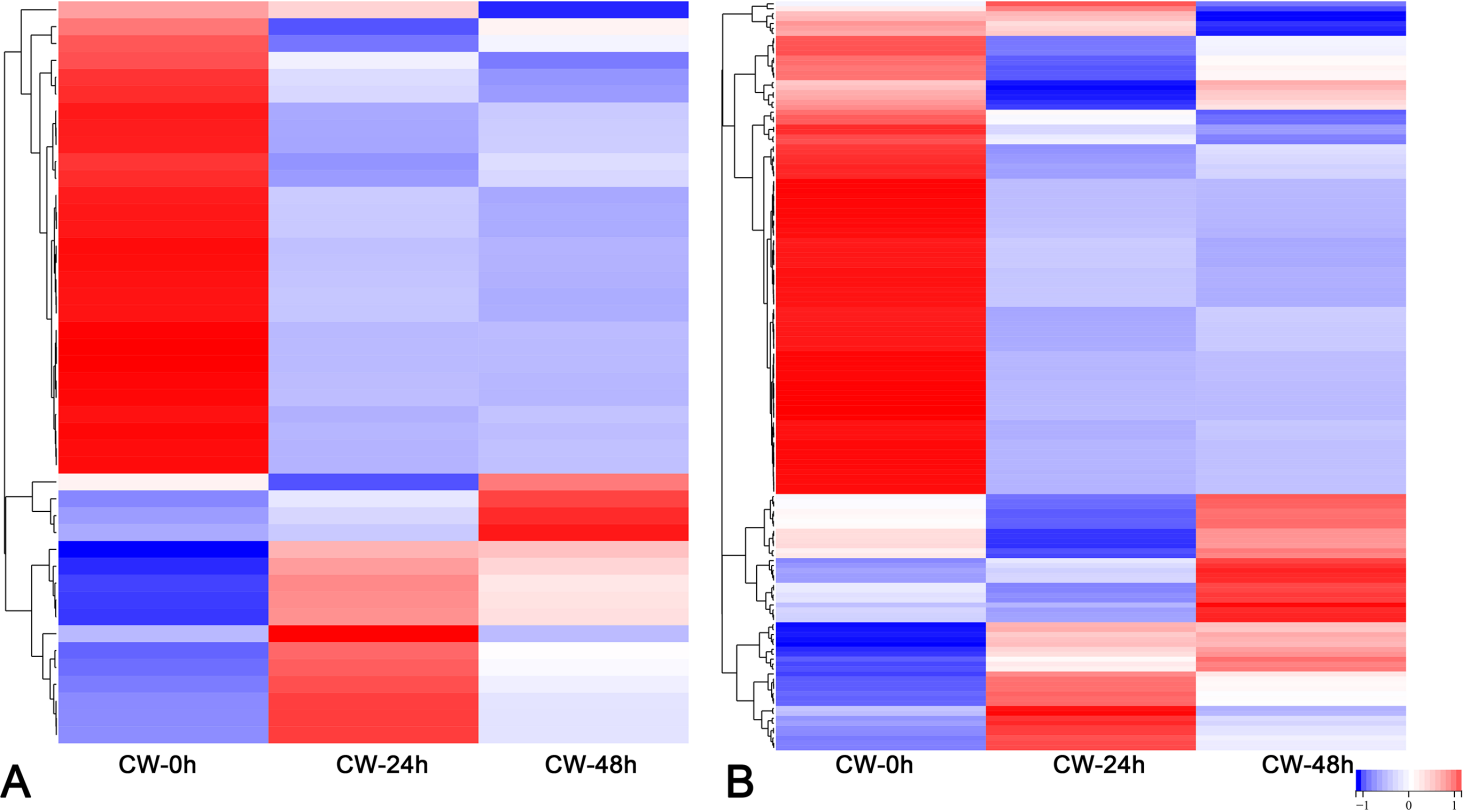
Expression patterns of DEGs. (A) The flavonoid biosynthesis pathway and (B) the phenylpropanoid biosynthesis pathway.

Flavonoids and phenylpropanoids play an important role in plant growth and development. As shown in Fig. 4A and Table S8, 28 DEGs in the flavonoid biosynthetic pathway were downregulated between CW-0 *vs.* CW-24, and this low level of expression persisted up to 48 h. Most of the DEGs which belong to caffeoyl-CoA O-methyltransferase downregulated after decapitation. There were 12 upregulated DEGs at 24 h after decapitation, which then decreased by 48 h. The DEGs of chalcone synthase upregulated after decapitation. Four DEGs were not upregulated until 48 h. In the phenylpropanoid biosynthesis pathway, there were 100 downregulated DEGs, 93 of which were downregulated at 24 h and seven were downregulated at 48 h. A total of 26 DEGs were significantly upregulated until 48 h. A large number of peroxidases were first downregulated and then upregulated after decapitation. Caffeoyl-CoA O-methyltransferase and caffeic acid 3-O-methyltransferase were expressed in large quantities before decapitation and downregulated rapidly after decapitation (Fig. 4B, Table S9).

### Analysis of DEGs in the plant hormone signal transduction pathway

**Figure 5:**
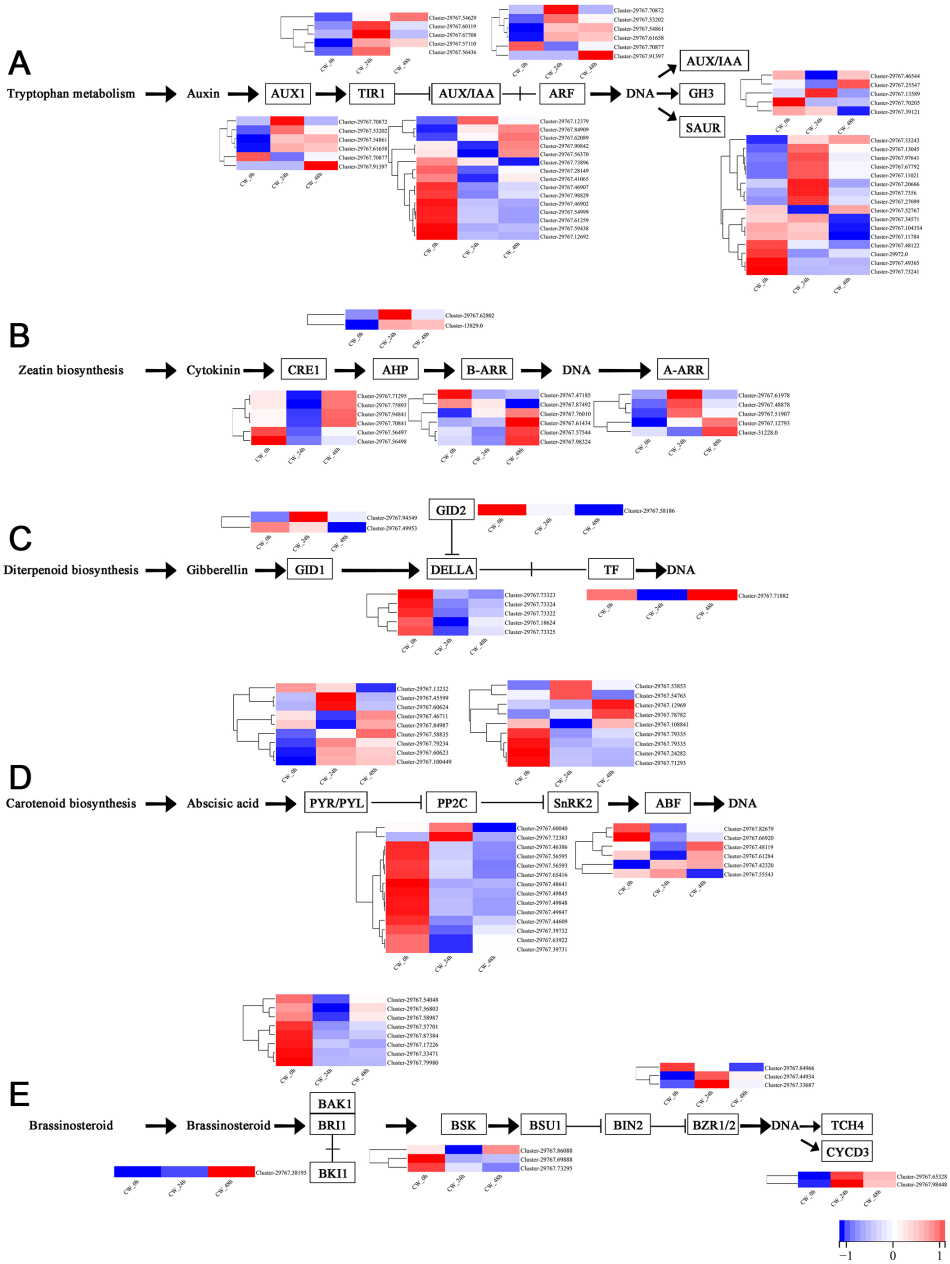
(A–E) The expression of DEGs in the plant hormone signal transduction pathway in CW-0, CW-24 and CW-48.

Plant hormones, including auxin, cytokinin, gibberellin, abscisic acid and brassinosteroid are involved in the germination and growth of axillary buds. In this study, the DEGs of these plant hormones in the signal transduction pathways were selected for analysis. In the auxin transduction pathway, most DEGs had similar expression patterns, with low expression 0 h and a significant increase 24 h after decapitation, then decreasing by varying degrees by 48 h. There were 10 AUX/IAA DEGs that were expressed at high levels when they were not decapitated but dropped to low levels of expression after decapitation (Fig. 5A). In the cytokinin transduction pathway, all six CRE1 DEGs were downregulated substantially followed by an increase 24 h after decapitation. The expression patterns of AHP and A-ARR DEGs were different from AUX/IAA and CRE1 DEGs the above; they were upregulated, then down-regulated after decapitation (Fig. 5B). In the GA transduction pathway, all five DELLA proteins were downregulated after decapitation(Fig. 5C). In the ABA pathway, PYR/PYL, PP2C, and SnRK2 all had a large number of DEGs. Most of the PP2C DEGs downregulated after decapitation (Fig. 5D). In the brassinosteroid pathway, the DEGs for both BAK1 and BSK were downregulated 24 h after decapitation, whereas two DEGs for CYCD3 were upregulated after decapitation (Fig. 5E).

**Figure 6:**
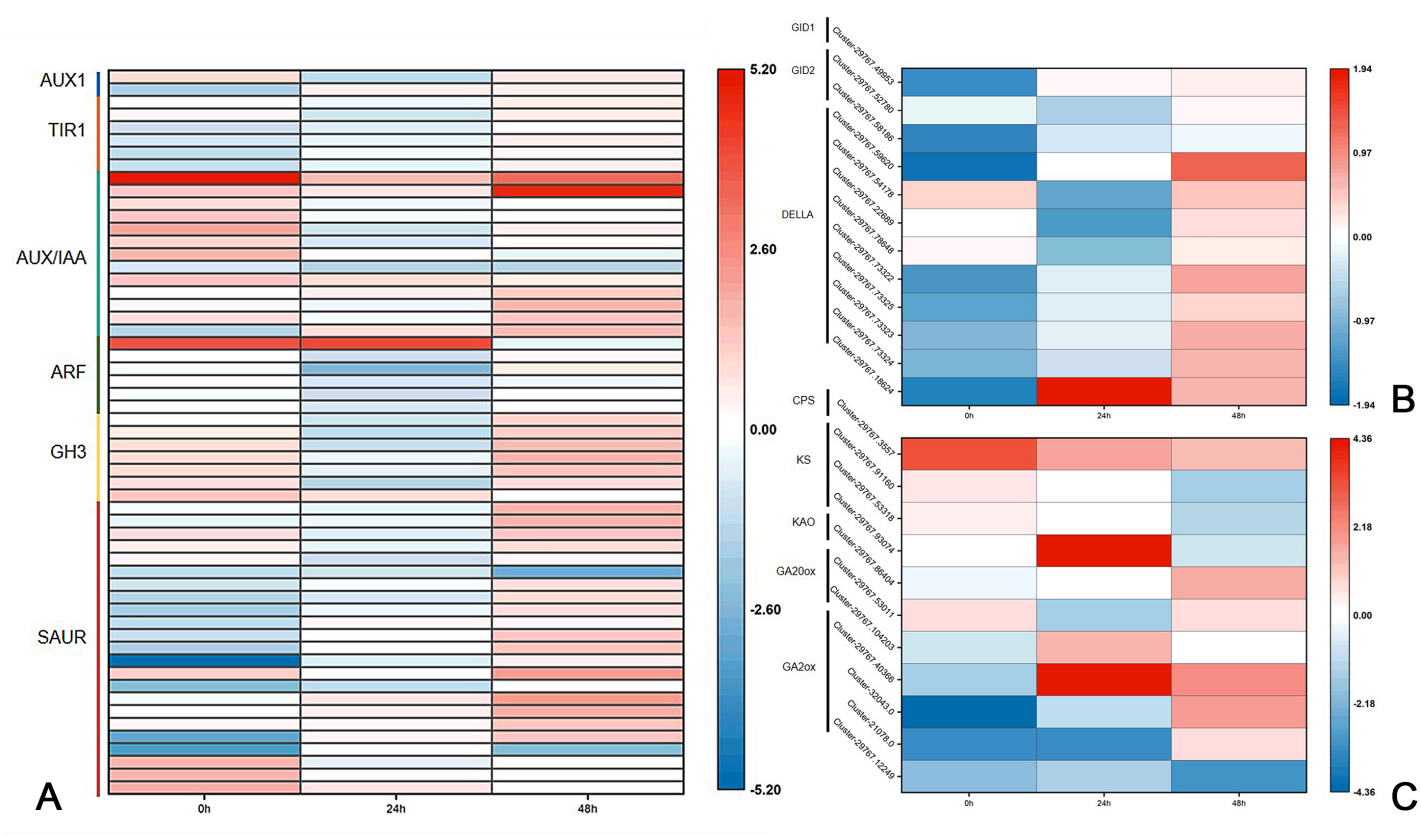
Differences of DEGs in CW-0 *vs.* C15-0, CW-24 *vs.* C15-24, and CW-48 *vs.* C15-48. (A) The auxin transduction pathway; (B) the GA transduction pathway; and (C) the diterpenoid biosynthesis pathway.

Previous research has shown that *DgLsL* may regulate the content of IAA and GAs, thus affecting the growth of plant axillary buds. For the auxin transduction pathway (Fig. 6A), C15 had more than 10 DEGs in both AUX/IAA and SAUR compared to CW, but the DEGs in AUX/IAA were upregulated and the DEGs in SAUR were more downregulated at 0 h. The DEGs in ARF and GH3 showed differential expression 24 h after decapitation, then returned to the 0 h state at 48 h. These results suggest that *DgLsL* responded rapidly after decapitation and affected the expression of genes in the auxin transduction pathway. In the GA transduction pathway, 12 DEGs were differentially expressed for GID1, GID2 and DELLA (Fig. 6B). Of these, compared to CW, nine DEGs were downregulated at 0 h and 24 h and 11 DEGs were upregulated at 48 h. There were 11 DEGs in the diterpenoid biosynthesis pathway (Fig. 6C). One CPS DEG was upregulated at 0, 24 and 48 h. DEGs expressions for GA2 was lower at 0 h, one DEG was upregulated 24 h after decapitation, and differential ploidy appeared to rise at 48 h. Compared with CW, most DEGs in C15 had lower expression in the GA pathway before decapitation. The expression of *DgLsL* was upregulated after decapitation, which may promote gene expression in the GA pathway affecting GA synthesis and transduction.

## Discussion

### Effect of plant hormones on axillary bud development

In recent years, the control of axillary buds by plant hormones has been intensively studied. Mutants of genes associated with meristem development have been analyzed in a range of plants including *Pisum sativum*, *Petunia* × *atkinsiana*, *Oryza sativa* L., *Nicotiana tabacum* L., and *Arabidopsis thaliana*, providing further evidence that shoot activation can be controlled by the auxin transporter-based system (Booker, Chatfield & Leyser, 2003; Sorefan et al., 2003; Snowden et al., 2005; Arite et al., 2007; Leyser, 2009). Plant hormones regulate the growth and development of plants universally, and the most important of these hormones is auxin, which was one of the first plant hormones to be discovered (Chatfield et al., 2000; Souza et al., 2003; Shi et al., 2019; Zhang et al., 2022). GAs belong to the diterpene family and are essential for the growth and development of plants. Previous studies have shown that a range of changes in endogenous auxin and GA levels are the key factors in regulating axillary bud development (Doebley, Stec & Hubbard, 1997; Chatfield et al., 2000; Nordström et al., 2004; Müller & Leyser, 2011). In this study, the KEGG pathway of the DEGs in the axillary buds of CW and C15 were analyzed 0 h, 24 h, and 48 h after decapitation. The results showed that several of the most enriched pathways in the different comparison groups included hormone signal transduction, phenylpropanoid biosynthesis, flavonoid biosynthesis, photosynthesis, and other pathways related to plant axillary bud growth.

In chrysanthemum with different branching types, auxin content plays an important role in apical dominance. The higher the auxin content in terminal buds, the stronger the inhibitory effect on the growth of axillary buds; the level of auxin in axillary buds also increase during activation (Hillman, Math & Medlow, 1977; Jiang et al., 2010). In *Phaseolus multiflorus* and *Ananas comosus*, the IAA content in the axillary buds increased significantly after decapitation. This increase in IAA content occurred before the increase in fresh weight of the lateral buds, suggesting that IAA may be related to axillary buds growths, and the increased of IAA content of axillary buds after decapitation may indicate the formation of new apical dominance (Gocal et al., 1991; Souza et al., 2003). These studies all showed that IAA content in axillary buds increased after decapitation. In the current study, most of the DEGs in the auxin transduction pathway had similar expression patterns; these genes had low expression levels before decapitation, significantly increase at 24 h, but decreased by varying degrees at 48 h. In CW, the sprouting of axillary buds after decapitation was inhibited by apical dominance, and the auxin pathway genes showed low expression levels. And the expression of the auxin pathway genes were up-regulated after the apical dominance of CW was removed, indicating that the axillary buds were gradually forming a new apical dominance. Similarly, in pea, the growth of axillary buds was induced 4–6 h after decapitation at all stem nodes (Morris et al., 2005). In chrysanthemum, 4–6 h after decapitation, only the auxin content in the stem close to the decapitated parts decreased, while the auxin content in the basal stems stayed similar to the non-decapitated plants. Axillary bud initiation precedes changes in auxin content and auxin transport, and this initiation is not inhibited by apically applied exogenous auxin, suggesting that decapitation triggers the early growth of axillary buds through mechanism not dependent on auxin. It is assumed that auxin acts after the axillary bud initiation stage to regulate apical dominance. GAs are the key hormones that regulate a range of activities during plant growth and development (Shi et al., 2019). At the leaf axils, axillary bud initiation requires low levels of GAs concentration (Zhang, 2018). In Arabidopsis, the gibberellin receptor mutant has a weak apical dominance and thus exhibits a multibranched phenotype (Koorneef et al., 1985). Many previous studies have shown that GAs inhibit axillary bud growth in Arabidopsis, *Oryza sativa* L., *Solanum lycopersicum* and *Liriodendron tulipifera* (Agharkar et al., 2007; Mauriat, Sandberg & Moritz, 2011). In this study, the average number of axillary buds in C15 was significantly higher than WT, and the gene expression of the GA pathway before decapitation was lower in C15 compared with CW, indicating that a low level of GAs in chrysanthemum could promote axillary bud sprouting, which is consistent with the results of previous studies.

### *DgLsL* promotes GA2ox synthesis

**Figure 7:**
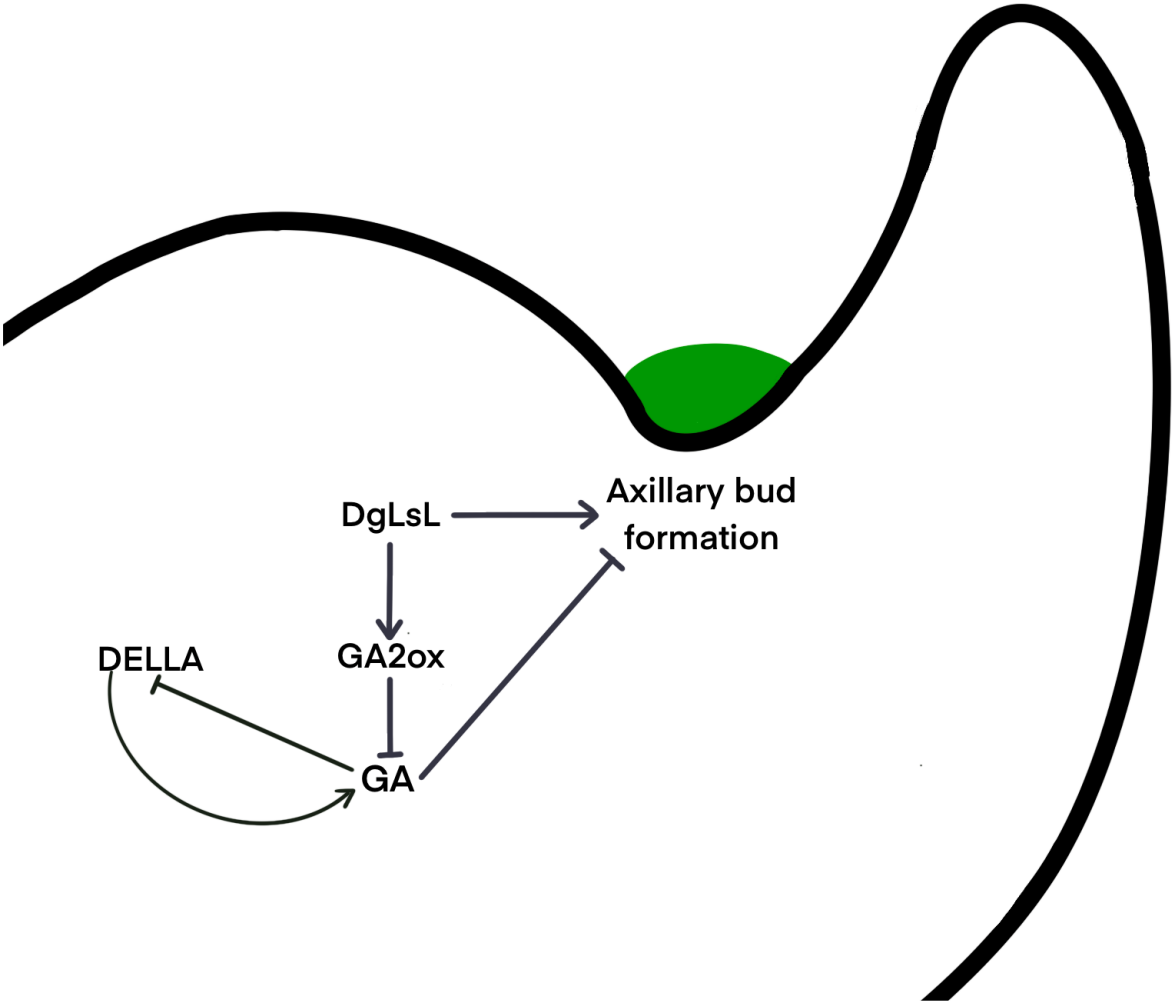
Hypothetical interaction network model of axillary bud emergence and development in chrysanthemum.

It is unclear whether different species use the same signaling network to determine the location of axillary meristem formation, or whether alternate pathways have been developed. Gibberellins may regulate axillary meristem formation through a complex network of regulation. The DELLA protein is a major blocker of the GA signaling pathway and is involved in a variety of physiological processes by interacting with many different transcription factors (Lee et al., 2002). It has been shown that DELLAs maintain GA homeostasis through feedback regulation of upstream positive regulators of the GA pathway (Sun, 2011) . Results here show that six pathways of the DELLA protein were upregulated in C15 in the 48 h after decapitation; three pathways were downregulated at 24 h and then upregulated at 48 h (Fig. 6B). It is hypothesized that in the ‘Jinba’ chrysanthemum, the DELLA protein inhibits GA expression and thus reduces GA content in axillary buds. A previous study transferred *DgLsL* sense and antisense genes into the ‘Jinba’ chrysanthemum variety, and the results showed that the introduction of the sense transformants significantly promoted branching, while the introduction of the antisense transformants significantly inhibited branching; GA3 content was lower in the terminal buds of plants that had the *DgLsL* sense gene introduced, and lower in the terminal buds of plants that had the antisense gene introduced. The GA3 content was lower in the terminal buds of the *DgLsL* plants and higher in the antisense *DgLsL* plants (Jiang et al., 2010). Later, the ‘Jinba’ chrysanthemum was treated with GA, 6-BA, IAA, ABA, and distilled water after decapitation. The expression of the *DgLsL* gene in the lateral buds responded differently to different hormone treatments after decapitation, with a more intense response to GA, and the expression of *DgLsL* in the lateral buds of ’Jinba’ reached about 4.5-fold at 24 h. GAs also have an inducing effect on the expression of the *DgLsL* gene (Luo et al., 2021). In the present study, the average number of sprouting axillary buds in C15 was 6.5-fold higher than in WT. Analysis of differential genes in the GA transduction pathway between wild-type plants and *DgLsL* overexpressing plants 0, 24 and 48 h after decapitation (Figs. 6B–6C) revealed that all four GA2-oxidase (GA2ox) pathways had significantly up-regulated expression. GA2ox is one of the key enzymes that affects gibberellin synthesis and metabolism. GA2ox expression was significantly up-regulated in C15, and GA2ox was able to break down GAs to reduce GA content in plants, and the number of axillary buds was higher in C15 than in WT. Previous studies have shown that overexpression of the GA catabolic gene GA2ox leads to an increase in the number of tillers or branches in rice, tomato, turfgrass and poplar (Agharkar et al., 2007; Lo et al., 2008; Mauriat, Sandberg & Moritz, 2011; Martínez-Bello, Moritz & López-Díaz, 2015). Therefore, it is hypothesized that low concentrations of GAs in chrysanthemum promote axillary bud germination. The *DgLsL* gene is a homolog of the *LAS* gene cloned in chrysanthemums, and may promote lateral bud germination by promoting GA2ox expression, reducing GA content n chrysanthemums (Fig. 7).

### Flavonoids involved in axillary bud development

Flavonoid metabolism is an important branch of phenylpropanoid metabolism (Hichri et al., 2011; Tohge et al., 2013), and flavonoids affect the development of lateral branches (Peer et al., 2004; Tan et al., 2019; Li et al., 2020; Shi et al., 2021). The overexpression of flavonoid biosynthesis pathway-related genes inhibits axillary bud growth in *A. thaliana*, whereas deletion of flavonoid biosynthesis pathway-related genes inhibits axillary bud growth (Borevitz et al., 2000; Brown et al., 2001). Low expression levels of genes related to the flavonoid biosynthetic pathway lead to a decrease in growth hormone transporter proteins in shoots and stems, reducing the transport rates of polar growth hormones (Lazar & Goodman, 2006). Flavonoids interact with regulatory proteins, promoting pin-formed (PIN) auxin efflux, and then flavonoid content increases at the sites of growth hormone accumulation (Peer et al., 2004). In this study, 28 DEGs in the flavonoid biosynthetic pathway were down-regulated in CW-0 *vs.* CW-24, and this low level of expression persisted up to 48 h. In the phenylpropanoid biosynthesis pathway, there were 100 downregulated DEGs down-regulated at different times. Most of the DEGs in the flavonoid biosynthesis and phenylpropanoid biosynthesis pathways were down-regulated at 24 h and 48 h, likely due to changes in auxin levels in the axillary buds after decapitation.

## Conclusion

This study performed a transcriptomic analysis of the axillary buds of CW and C15 of ‘Jinba’ chrysanthemum 0 h, 24 h and 48 h after decapitation. The analysis showed that the genes of the flavonoid, phenylpropanoids and plant hormone pathways appeared to be differentially expressed, indicating their important roles in axillary bud germination. The results also showed that auxin acts after the axillary bud initiation stage to regulate apical dominance, affecting axillary buds growth. In contrast, gibberellin and auxin have an antagonistic relationship and jointly regulate the germination of axillary buds. A comparison of C15 with CW showed that *DgLsL* overexpression resulted in more axillary buds and fewer nodes in chrysanthemum, indicating that there are many expression changes of genes related to axillary bud development that would weaken the effect of *DgLsL* after decapitation. GA inhibits DELLA protein expression and DELLA negatively regulates GA. *DgLsL* promotes GA2ox expression and GA2ox breaks down GA, thus reducing the GA content in axillary buds. It is hypothesized that low concentrations of GA in chrysanthemums promote axillary bud germination. The results of this confirmed the results of previous studies on axillary bud germination and growth and revealed important roles of genes involved in plant hormone biosynthesis and signal transduction, which help illuminate the gene patterns involved in axillary bud germination and growth.

## Supplemental Information

10.7717/peerj.16436/supp-1 Supplemental Information 1 Statistical chart of KEGG metabolic pathway classification The vertical coordinate of the graph is the name of the KEGG metabolic pathway, and the horizontal coordinate is the number of genes annotated to the pathway and their number as a percentage of the total number of genes annotated on it.

10.7717/peerj.16436/supp-2 Supplemental Information 2 GO pathway enrichment scatter plot The vertical axis indicates the pathway name, the horizontal axis indicates the GO term corresponding to the Rich factor, the size of qvalue is indicated by the color of the dot, the smaller the qvalue the closer the color is to red, the number of differential genes contained under each pathway is indicated by the size of the dot.

10.7717/peerj.16436/supp-3 Supplemental Information 3 Primers of qRT-PCR

10.7717/peerj.16436/supp-4 Supplemental Information 4 RT-PCR system

10.7717/peerj.16436/supp-5 Supplemental Information 5 RT-PCR procedure

10.7717/peerj.16436/supp-6 Supplemental Information 6 QRT-PCR procedure

10.7717/peerj.16436/supp-7 Supplemental Information 7 Sample sequencing data evaluation statistics table

10.7717/peerj.16436/supp-8 Supplemental Information 8 Statistical table of assembly results

10.7717/peerj.16436/supp-9 Supplemental Information 9 Annotation results of different databases

10.7717/peerj.16436/supp-10 Supplemental Information 10 The FPKM of DEGs in Phenylpropanoid biosynthesis

10.7717/peerj.16436/supp-11 Supplemental Information 11 The FPKM of DEGs in Flavonoid biosynthesis

10.7717/peerj.16436/supp-12 Supplemental Information 12 Raw data

## Additional Information and Declarations

### DNA Deposition

The following information was supplied regarding the deposition of DNA sequences: The sequence is available at GenBank: AB206874.1.

### Data Availability

The following information was supplied regarding data availability: The raw measurements are available in the Supplementary File.
