# Supplementary figures and images for: Transcriptome analysis during axillary bud growth in chrysanthemum (chrysanthemum×morifolium)
Source: PeerJ. 2023 Dec 15;11:e16436. doi: 10.7717/peerj.16436 (PMC10726743; doi:10.7717/peerj.16436)

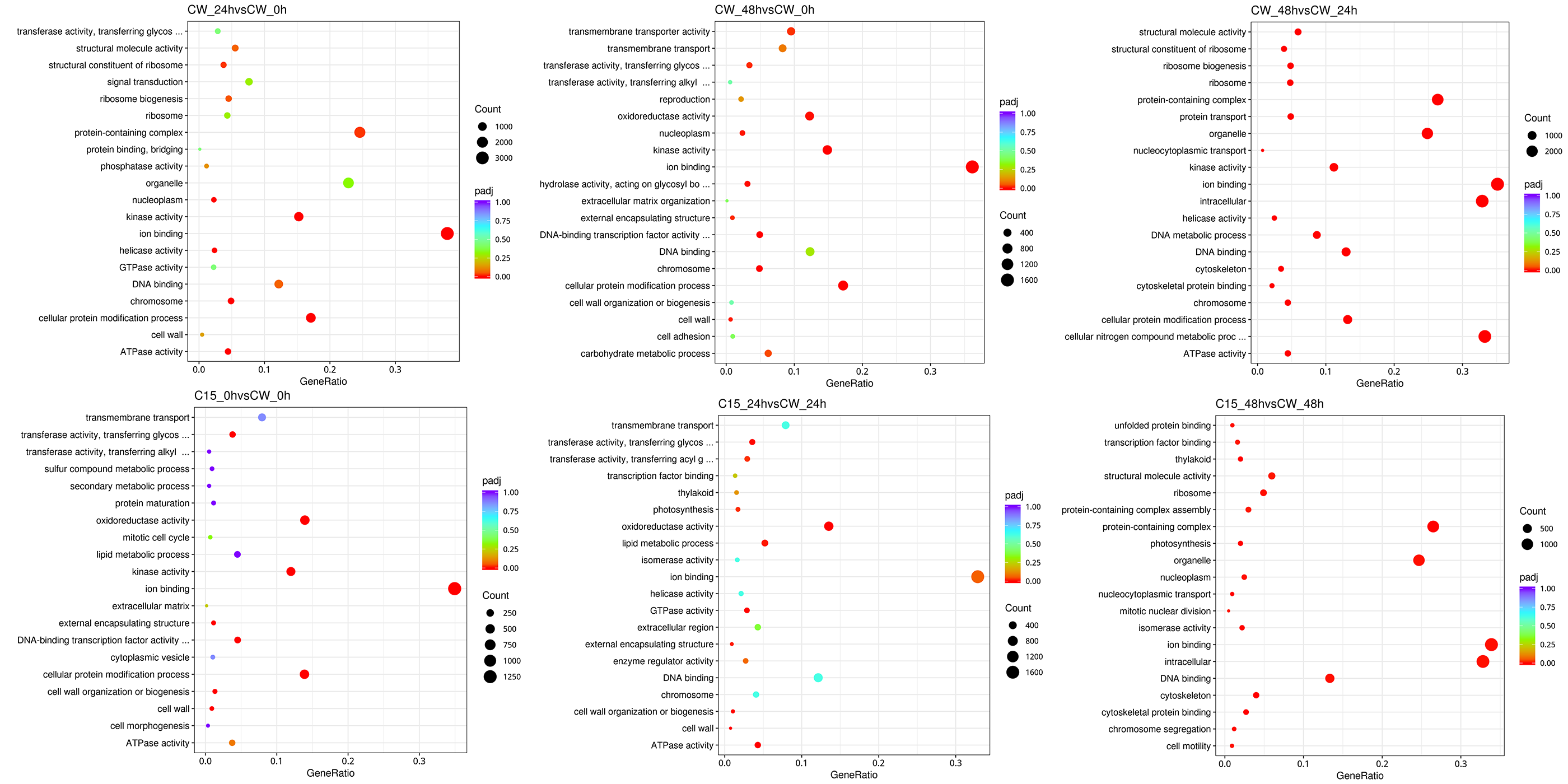

Supplement: Supplemental Information 1 — The vertical coordinate of the graph is the name of the KEGG metabolic pathway, and the horizontal coordinate is the number of genes annotated to the pathway and their number as a percentage of the total number of genes annotated on it. [file peerj-11-16436-s001.png]

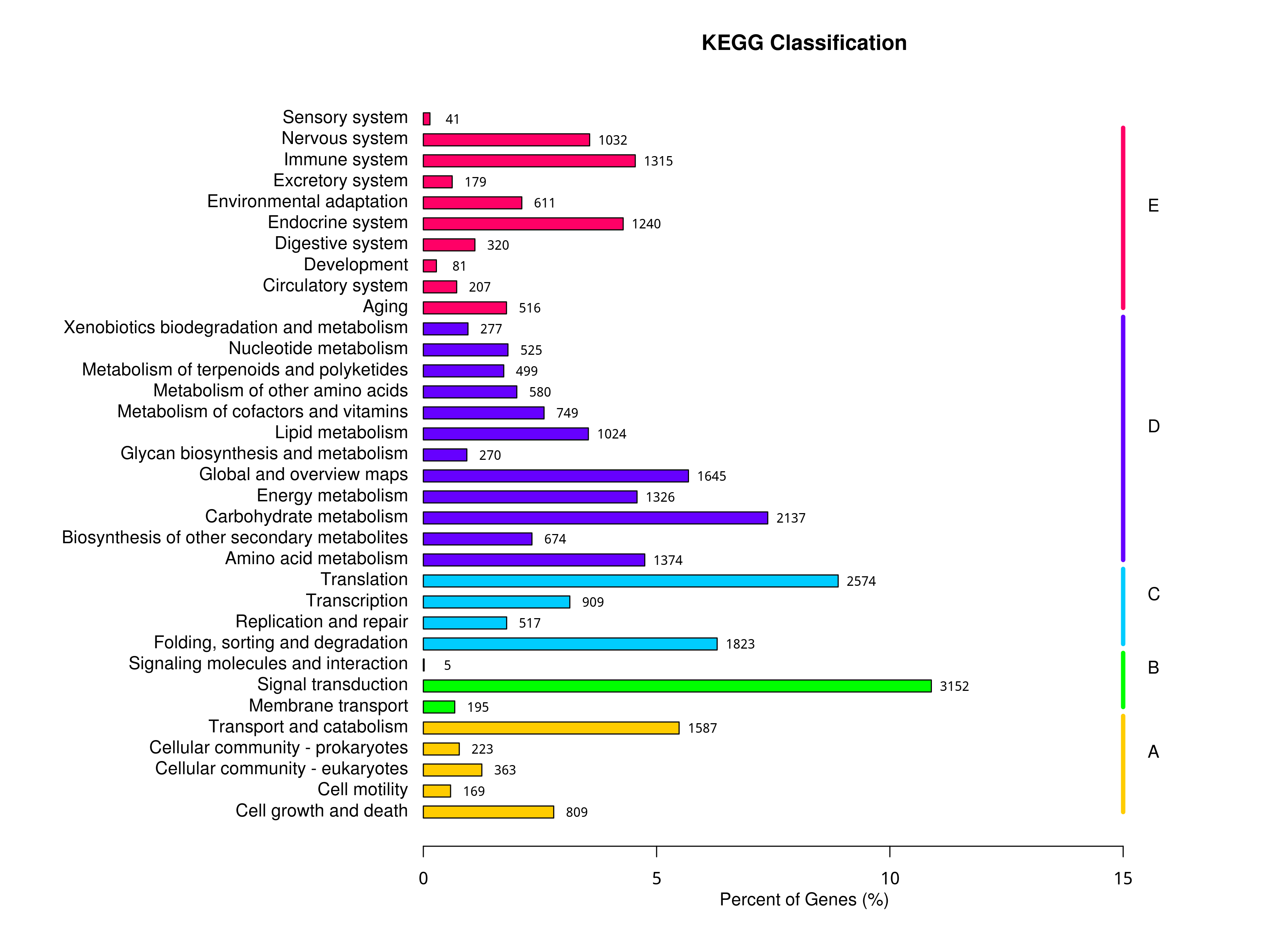

Supplement: Supplemental Information 2 — The vertical axis indicates the pathway name, the horizontal axis indicates the GO term corresponding to the Rich factor, the size of qvalue is indicated by the color of the dot, the smaller the qvalue the closer the color is to red, the number of differential genes contained under each pathway is indicated by the size of the dot. [file peerj-11-16436-s002.png]
